# Novel method for site-specific induction of oxidative DNA damage reveals differences in recruitment of repair proteins to heterochromatin and euchromatin

**DOI:** 10.1093/nar/gkt1233

**Published:** 2013-11-29

**Authors:** Li Lan, Satoshi Nakajima, Leizhen Wei, Luxi Sun, Ching-Lung Hsieh, Robert W. Sobol, Marcel Bruchez, Bennett Van Houten, Akira Yasui, Arthur S. Levine

**Affiliations:** ^1^Department of Microbiology and Molecular Genetics, University of Pittsburgh School of Medicine, Pittsburgh, PA 15213, USA, ^2^University of Pittsburgh Cancer Institute, University of Pittsburgh School of Medicine, 5117 Centre Avenue, Pittsburgh, PA 15213, USA, ^3^School of Medicine, Tsinghua University, No.1 Tsinghua Yuan, Haidian District, Beijing 100084, People’s Republic of China, ^4^Department of Pharmacology & Chemical Biology, University of Pittsburgh School of Medicine, Pittsburgh, PA 15213, USA, ^5^Department of Human Genetics, University of Pittsburgh Graduate School of Public Health, Pittsburgh, PA 15213, USA, ^6^Department of Chemistry and Department of Biological Sciences, Carnegie Mellon University, Pittsburgh, PA 15213, USA and ^7^Division of Dynamic Proteome, Institute of Development, Aging, and Cancer, Tohoku University, Seiryomachi 4-1, Sendai 980-8575, Japan

## Abstract

Reactive oxygen species (ROS)-induced DNA damage is repaired by the base excision repair pathway. However, the effect of chromatin structure on BER protein recruitment to DNA damage sites in living cells is poorly understood. To address this problem, we developed a method to specifically produce ROS-induced DNA damage by fusing KillerRed (KR), a light-stimulated ROS-inducer, to a tet-repressor (tetR-KR) or a transcription activator (TA-KR). TetR-KR or TA-KR, bound to a TRE cassette (∼90 kb) integrated at a defined genomic locus in U2OS cells, was used to induce ROS damage in hetero- or euchromatin, respectively. We found that DNA glycosylases were efficiently recruited to DNA damage in heterochromatin, as well as in euchromatin. PARP1 was recruited to DNA damage within condensed chromatin more efficiently than in active chromatin. In contrast, recruitment of FEN1 was highly enriched at sites of DNA damage within active chromatin in a PCNA- and transcription activation-dependent manner. These results indicate that oxidative DNA damage is differentially processed within hetero or euchromatin.

## INTRODUCTION

Reactive oxygen species (ROS) can be generated endogenously during cellular respiration or in response to infection and exogenously by chemical and physical agents. ROS mainly induce oxidized bases and single-strand breaks (SSBs) in DNA. These lesions are repaired via the base excision/SSB repair (BER/SSBR) pathways (1,2). If left unrepaired, ROS-induced damage blocks DNA replication and transcription, leading to genome instability and genetic alterations that can result in mutations that in turn drive tumorigenesis. In BER, DNA glycosylases remove the damaged base, followed by AP endonuclease to introduce a nick in the DNA strand (3). In SSBR, activation of poly(ADP-ribose)polymerase 1 (PARP1) plays a central role (4,5). PARP1 is involved in the poly(ADP)-ribose (PAR)-modification of histones and DNA repair proteins. In recent years, PARP inhibitors (PARPi) have been developed for use in cancer therapy (6,7). XRCC1, a scaffold protein that accumulates at sites of SSBs in association with PAR, is necessary for repair progression as it recruits other repair factors (4). Both BER and SSBR are carried out with short-patch or long-patch repair synthesis by DNA polymerases and completed with ligation by DNA ligase III or DNA ligase I. DNA polymerase ß (Polß), which contains an N-terminus dRP lyase domain and a C-terminal polymerase domain, is involved in both short- and long-patch BER (8,9).

ROS-induced DNA damage is repaired in living cells within a temporal and spatial context, and chromatin structure is critical to a consideration of DNA repair processes *in situ* (10). DNA is wrapped around histones to form a mononucleosome structure, and nucleosomes are further condensed to form chromatin structures in cells. *In vitro* studies using reconstituted nucleosomes containing rotationally positioned uracil indicate that the catalytic activity of BER enzymes is suppressed when working on damage in the context of chromatin (10); furthermore, the ATPase chromatin remodeling factor SWI/SNF shows a very weak effect on 8-oxoG BER removal (11), indicating the importance of chromatin remodeling in facilitating BER. To date, there has been no method to induce site-specific oxidative DNA damage, especially base modifications, in living cells. Therefore, it is not known whether the DNA repair mechanisms associated with base damage differ within active or condensed chromatin.

In the work reported here, we used a hydrozoan derived fluorescent protein, KillerRed (KR), to produce ROS-induced oxidative DNA damage in defined genome locations within living cells (12). It also has been reported that KR induces strong cytotoxicity through the chromophore-assisted light inactivation effect (12–14). Crystallographic analysis of KR in its native and bleached states demonstrates how its structure facilitates the formation of oxygen radicals and superoxide through the excited chromophore (15,16). While superoxide cannot damage DNA, it can spontaneously (or through CuZnSOD), form hydrogen peroxide which will then, in the presence of metal cations such as Fe^2+^ and/or Cu^+^, induce base damage and DNA SSBs. For example, it has been previously shown that cells expressing KR fused to histone H2A or H2B showed light-induced blockage of cell division and increased DNA strand breaks (17,18) and KR caused cell toxicity both *in vitro* as well as *in vivo*, resulting in enhanced tumor cell death (19).

Based on the underlying mechanism of ROS generation from activated KR (after exposure to visible light), we developed an experimental system for visualizing the recruitment of BER enzymes in real time at a specific locus by expressing and localizing tetR-KR or TA-KR to a defined 90-kb site of condensed or open chromatin, respectively, in human cells. We found that light irradiation of cells expressing KR induces oxidative DNA damage at a specific genomic site, making it possible to characterize the recruitment of proteins involved in the oxidative repair pathway in live cells within sites of condensed or open chromatin.

## MATERIALS AND METHODS

### Cell lines, transfection and chemicals

U2OS TRE cell line was described before as U2OS SCE 19 (20). In this cell line, 200 copies of pTRE/I-SceI were integrated in U2OS cells. Another U2OS 263 (a control cell line harboring 200 copies of the TRE element) (21) was used. Both cell lines were cultured in DMEM with 10% FBS at 37°C. Plasmids were transfected with Fugene-6 (Life Technology). The PARP inhibitors PJ34 (Sigma) or Olaparib (Sigma) were used. ROS scavengers NAC (500 mM/NaOH) (*N*-acetylcysteine) (Sigma), and MnTBAP (Enzo Life Science) were used. NAC (500 mM in NaOH) treatment was used with a 20 mM final concentration, and MnTBAP (100 mM/DMSO) was used with a 100 µM final concentration in PBS for 1 h. H_2_O_2_ (Sigma) treatment was used at the indicated concentration in PBS for 1 h. siFEN1 to target the UTR region (Thermo Scientific, A-010344-14) and siPARP1 (Thermo Scientific, E-006656-00) were transfected with DharmaFECT transfection regent (Thermo Scientific, T-2001-02). RNA polymerase II inhibitor 5,6-dichloro-1 -β-d-ribofuranosylbenzimidazole (DRB) (Sigma) stock concentration is 20 mM; final concentration used is 20 µM, added to cells for 24 h before irradiation or other treatment.

### FISH assay

U2OS/I-SceI-TRE-19 cells were cultured with thymidine (300 µg/ml) for 16 h, washed three times with PBS, and further cultured for 6 h without and 4 h with the addition of Colcemid (0.02 µg/ml) before fixation with methanol/acetic acid (3:1). The pTRE-I-SceI plasmid was labeled with Red-dUTP (Abbott) and hybridized with a chromosome marker probe overnight. Hybridized chromosome samples were washed and signals were detected by fluorescence microscopy.

### ChIP assay

U2OS TRE cells were plated in a 10-cm dish at ∼25% confluency and incubated overnight. The cells were transfected with tetR-KR by Lipofectamine 2000 (Life Technologies) and incubated overnight. The cells were harvested and prepared for immunoprecipitation by using a ChIP-IT express enzymatic kit (active motif). Anti-phospho-histone H2AX (Ser139) (Millipore) was used for IP. Specific DNA sequences present in the immune precipitates were amplified by the following primers; CMV forward, TGT ACG GTG GGA GGC CTA TAT AA; pEGFP N1 primer, CGT CGC CGT CCA GCT CGA CCA G. The length of amplified DNA fragment is ∼220 bp.

### Cell survival (MTT and colony formation assays)

A total of 2 × 10^5^ U2OS TRE cells per 60-mm Petri dish were transfected with siFEN1 (72 h before assay), WT-FEN1, or FEN1-FF343/344AA (48 h before assay). A total of 5000 cells were seeded into 96-well plates; 48 h after preparation, cells were treated with H_2_O_2_ (Sigma) at the indicated concentration for the MTT assay (Promega).

### EU incorporation

Using Click-iT® RNA Imaging Kits (Invitrogen), we prepared a 2 mM working solution of EU and added an equal volume of this 2× EU working solution to the media containing cells before light irradiation. After light irradiation for 10 min, cells were incubated under normal cell culture conditions for 1 h, followed immediately by cell fixation, permeabilization and immunostaining for detection.

### Flow cytometry

Cells were trypsinized, collected and then resuspended in 5 ml of ice cold 70% ethanol overnight. After washing with 5 ml PBS containing 2% FBS, the cell pellets were incubated with 0.2 ml of PBS containing 2% FBS, 10 µl of 1 mg/ml PI and 2 µl of 10 mg/ml RNaseA for 30 min at 3°C in the dark. Cells were counted by using a BD Accuri C6 flow cytometer for analysis of the cell-cycle profile.

### Plasmids

KR or mCherry was amplified by PCR with additional BamHI and EcoRI sites and subcloned into a pBROAD3 tetR-ICP22 NLS vector (22) to yield pBROAD3/tetR-KR and pBROAD3/tetR-mCherry. pBROAD3/TA-KR was made by inserting tetR+VP16 sequences into the AgeI and BamHI sites in pBROAD3/tetR-KR. VP16 sequences were amplified by 5′ TA_AgeI: gcg acc ggt atg gca tct aga tta gat aaa ag and 3′TA+NLS_BamHI: gcg gga tcc cg TAC CTT TCT CTT CTT TTT TGG ATC cgg ccc acc gta ctc gtc aat tc, based on the template of pCherry-TA-ER (23). Human cDNAs containing full-length XRCC1, DNA Polß and PCNA were described before (4). The cDNA of PARP1, NeiL1, NeiL2, FEN1, all deletions and mutants of XRCC1, Polß and FEN1 were amplified with SalI/XhoI and NotI and subcloned into pEGPF-C1 (Clontech). YFP-RNA Polymerase II was provided by Dr Susan Janicki; myc-cdt1 was provided by Dr Chun Liang; GFP-SMUG1 was provided by Dr Samuel Wilson.

### Microscopy

**Figure 1.**
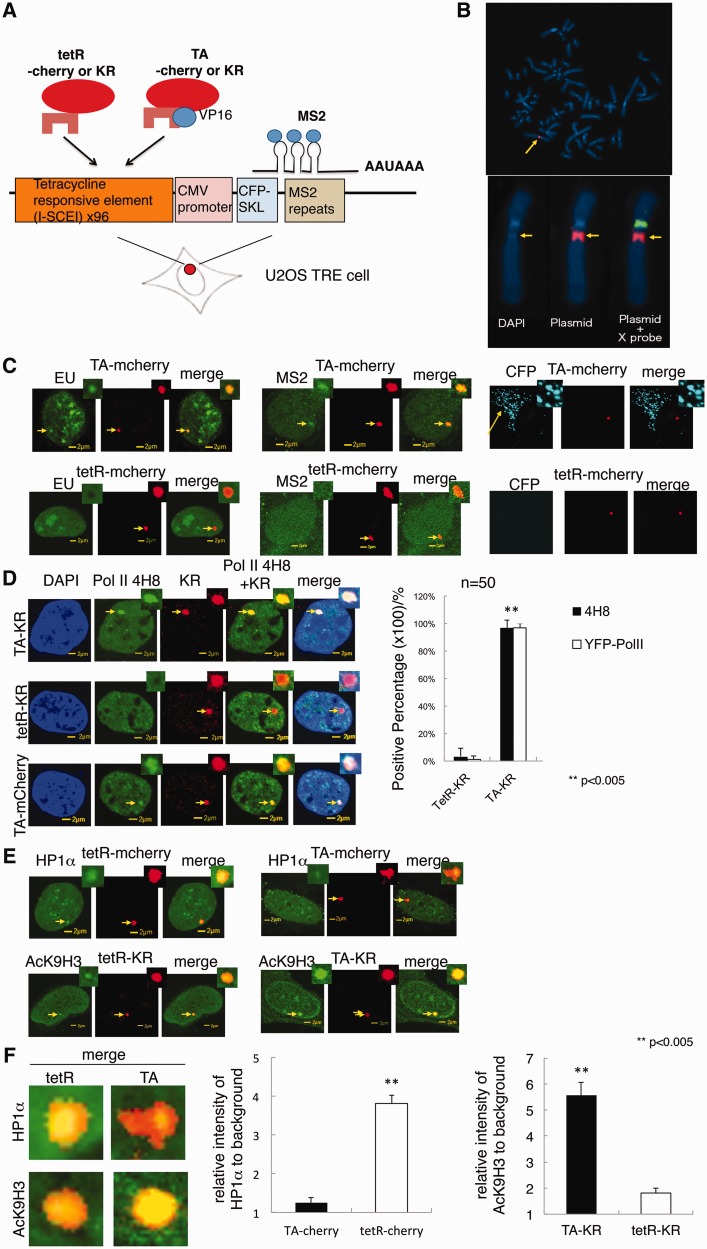
Chromatin status at site-specific genomic DNA by targeting tetR-KR or TA-KR to an integrated TRE site. (**A**) Scheme of tetR- and TA-tagged KR expression in the U2OS TRE cell line. To induce ROS-mediated damage at a specific locus in the genome, we fused KR to the tetracycline repressor (tetR) to induce ROS damage in a 90-kb TRE array (total of 96 repeats) in U2OS cells. In the cell line, 96 random TRE repeats were integrated into the genome site in U2OS cells at ∼200 copies. The plasmid also contains 24 tandem MS2 bacteriophage viral replicase translational operators (MS2 repeats) and SKL-tagged CFP after the TRE array and CMV promoter. (**B**) FISH assay of the U2OS TRE cell line. Blue is DAPI staining. Red is staining of the TRE region. Green is the X-chromosome specific probe. (**C**) Incorporation of EU, translocation of GFP-MS2 and expression of SKL-CFP were monitored in U2OS TRE cell line 24 h after transfection with tetR or TA tagged mcherry. (**D**) U2OS TRE cells transfected with either tetR or TA were stained with phospho-RNA Pol II antibody 4H8, which specifically recognizes pSer5 of the CTD heptapeptide of RNA Pol II. Quantification of localization of 4H8 staining and recruitment of YFP Pol II at the site of tetR and TA–KR in U2OS TRE cells transfected with tetR-KR or TA-KR is shown. (**E**) Localization of GFP-HP1α in U2OS TRE cells transfected with either tetR-cherry or TA-cherry (upper panel). U2OS TRE cells transfected with either tetR-KR or TA-KR were fixed and stained with H3AcK9 (lower panel). (**F**) Right panel shows the magnification of colocalization of HP1α and H3AcK9 at the site of tetR or TA. The intensity of HP1α and AcK9H3 at the site of tetR and TA was quantified. Mean value with SD is the intensity in 10 cells.

**Figure 2.**
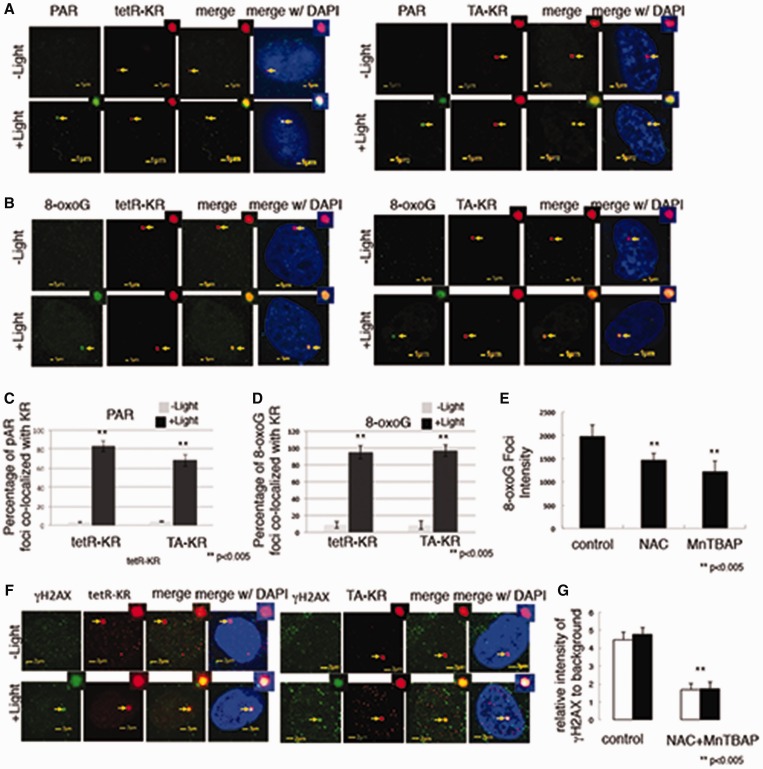
Site-specific genomic DNA damage by targeting tetR-KR or TA-KR to an integrated TRE site. (**A** and **B**) U2OS TRE cells transfected with tetR or TA–KR with or without exposure to a 15-W SYLVANIA cool white fluorescent bulb for 10 min were stained after treatment with anti-PAR (A), anti-8-oxoG (B) and anti-KR (−Light: without light activation, +Light: with light activation). (**C** and **D**) Graph shows the percentage of colocalization between PAR, oxoG and KR in 50 cells. Mean values with a SD in three independent experiments are given in all of the following quantification graphs. The *P*-value is calculated by Student’s *t*-test using stat plus software; *P *< 0.005 is shown as double asterisks. (**E**) U2OS TRE cells transfected with tetR-KR were treated with NAC (20 mM) or MnTBAP (100 µM) immediately before exposure to a 15-W SYLVANIA cool white fluorescent bulb for 10 min. Cells were fixed and stained with anti-8-oxoG after light activation. The intensity of 8-oxoG (average of 10 cells) at the site of tetR was quantified. SD is the intensity of 8-oxoG in 10 cells. (**F**) U2OS TRE cells transfected with tetR or TA–KR with or without exposure to a 15-W SYLVANIA cool white fluorescent bulb for 10 min were stained after treatment with anti-γH2AX and KR (−Light: without light activation, +Light: with light activation). (**G**) U2OS TRE cells were treated with NAC (20 mM) and MnTBAP (100 µM) immediately before exposure to a 15-W SYLVANIA cool white fluorescent bulb for 10 min. Cells were fixed and stained with anti-γH2AX after light activation. The intensity of γH2AX at the site of tetR was quantified. Mean value with SD is the intensity of 8-oxoG in 10 cells.

**Figure 3.**
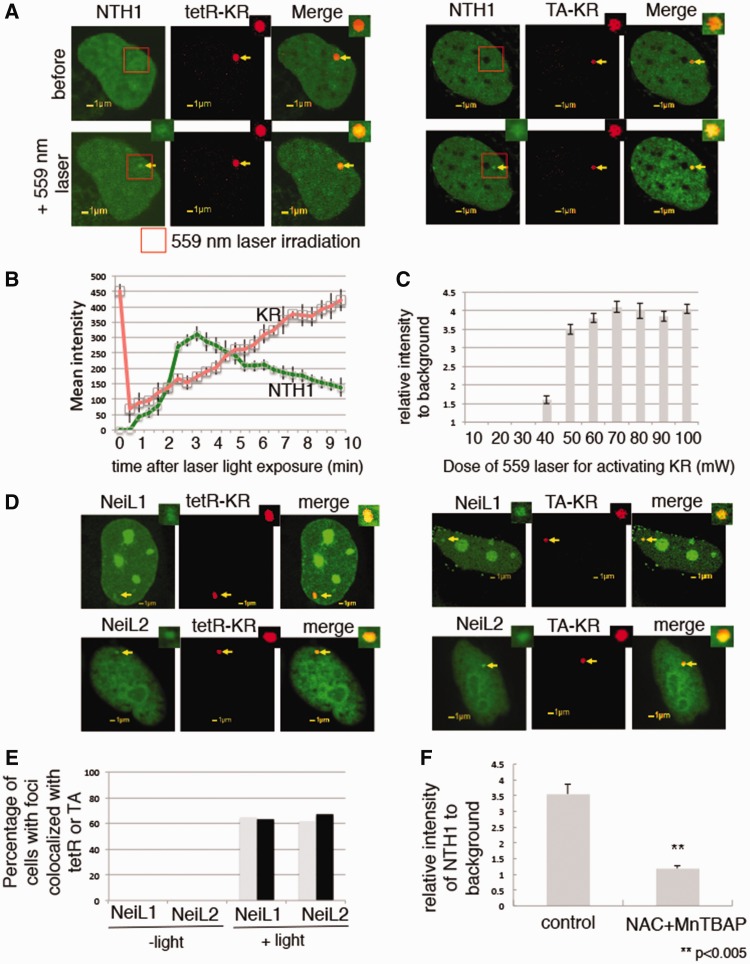
KR activation with either light or 559-nm laser excitation efficiently induces recruitment of DNA glycosylases. **(A**) Damage response of NTH1 to the site of tetR-KR (left) or TA-KR (right)-induced DNA damage 3 min after 559 nm laser (50 mW) bleaching. The red rectangle shows the selected bleaching area of the 559-nm laser. (**B**) Quantification of kinetics of GFP-NTH1 (green) and tetR-KR (red) at the site of damage. The mean intensity of each data point was obtained after subtraction of the background intensity in the irradiated cell. (**C**) Quantification of damage response of GFP-NTH1 3 min after the indicated laser light activation of tetR-KR. The fold increase was obtained from the foci intensity/the background intensity at the site of KR. Data are mean values with a SD of three cells. (**D**) Damage response of NEIL1 and NEIL2 to the site of tetR (left) or TA (right)—KR-induced DNA damage with the exposure to a 15-W SYLVANIA cool white fluorescent bulb for 10 min. (**E)** Quantification of percentage of cells with colocalization of GFP-NEIL1 or NEIL2 at the site of tetR or TA before and after a 15-W SYLVANIA cool white fluorescent bulb for 10 min. (**F**) U2OS TRE cells were treated with NAC (20 mM) and MnTBAP (100 µM) immediately before exposure to a 15-W SYLVANIA cool white fluorescent bulb for 10 min. Cell were fixed in 5-min light activation. The relative intensity of NTH1 at the site of tetR (intensity of NTH1 at the site of tetR/intensity of NTH1 area away from tetR) was quantified. Mean value with SD is the relative intensity of NTH1 in 10 cells.

**Figure 4.**
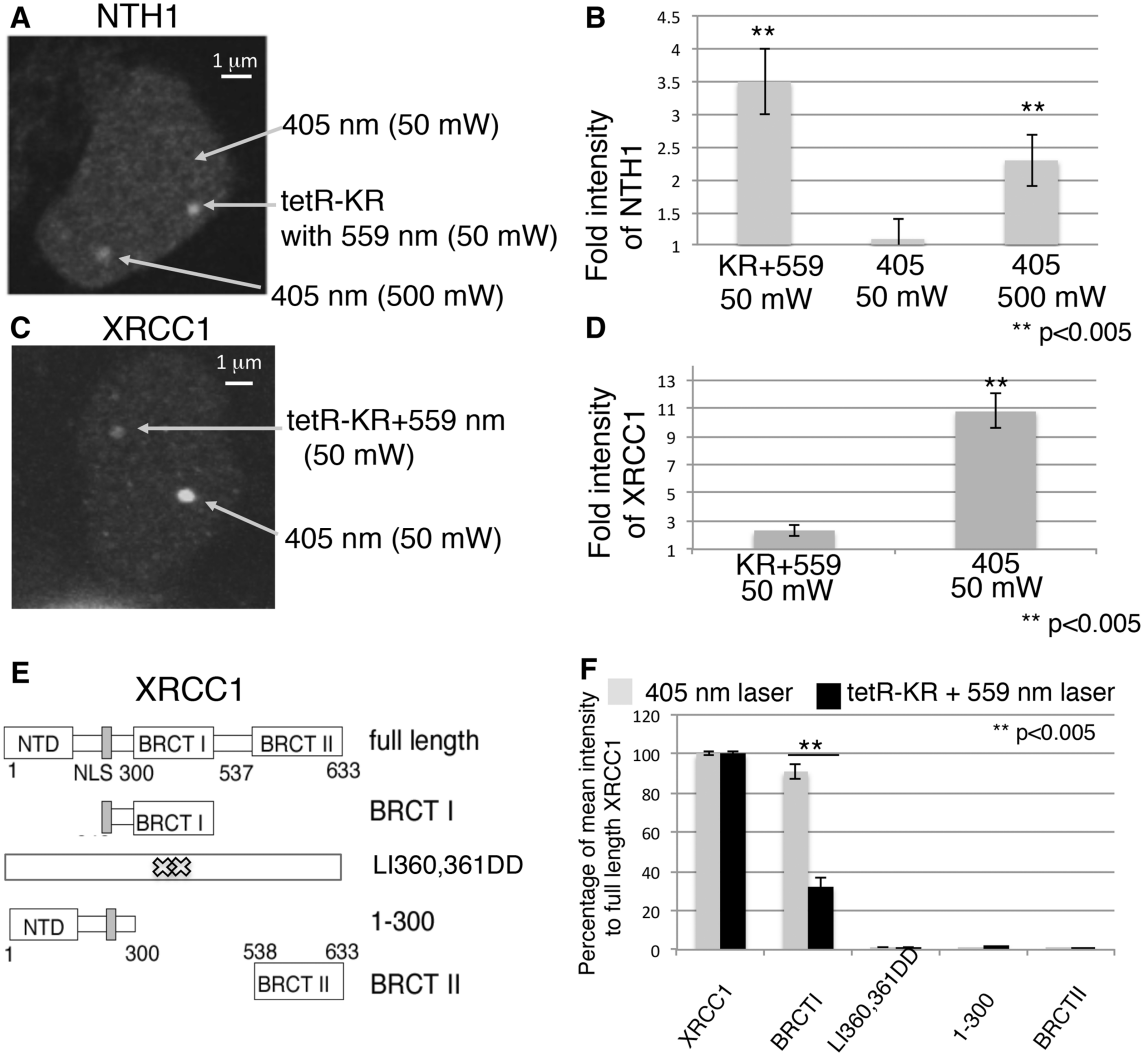
Comparison of the recruitment of repair factors to KR plus light-induced damage and 405-nm laser-induced damage. (**A**) GFP-NTH1 and tetR-KR were expressed in the U2OS TRE cell line. KR was bleached at full power 559-nm laser light for 50 scans with a rate of 1 mW/scan (total 50 mW); another indicated point in the same cell was irradiated with full power 405-nm laser light for 10 scans with a rate of 5 mW/scan (total 50 mW). (**B**) The quantification of damage response of GFP-NTH1 at the site of KR-induced damage or 405-nm laser-induced damage 3 min after irradiation. The fold increase was obtained from foci intensity/background intensity. Mean values with a SD of 10 cells are given. (**C** and **D**) The damage response of GFP-XRCC1 under the same conditions as in (A) and quantification of the damage response of GFP-XRCC1 at the site of KR-induced damage or 405-nm laser-induced damage 3 min after irradiation. (**E**) Schematic of deletion mutants of XRCC1 used in this study. (**F**) Quantification of the damage response ability of domains of XRCC1 relevant to full-length XRCC1. The average mean intensity of each domain (BRCT I, LI360/361DD, 1-300NTD, BRCT II)/mean intensity of full-length XRCC1 in response to laser-induced SSBs (gray) or KR induced base damage (black) is shown. Mean values with a SD of three independent experiments.

**Figure 5.**
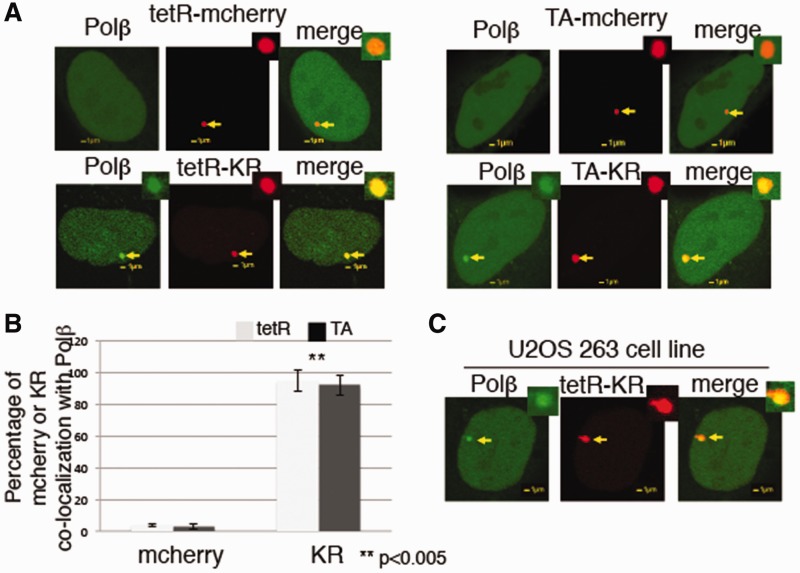
KR activation induces recruitment of DNA Polß at the sites of both tetR and TA in U2OS TRE and U2OS 263 cell lines. (**A**) Damage response of Polß to the site of tetR or TA-fused mCherry or KR 3 min after 559-nm laser light irradiation in U2OS TRE cell line. (**B**) Graph shows the percentage of colocalization between Polß and tetR and TA-fused mCherry (left), Polß and tetR or TA-fused KR (right) in 50 cells in U2OS TRE cell line. Mean values with a SD of three independent experiments. (**C**) The recruitment of Polß at the site of tetR-KR in U2OS (263) cell line 3 min after 559-nm laser bleaching.

**Figure 6.**
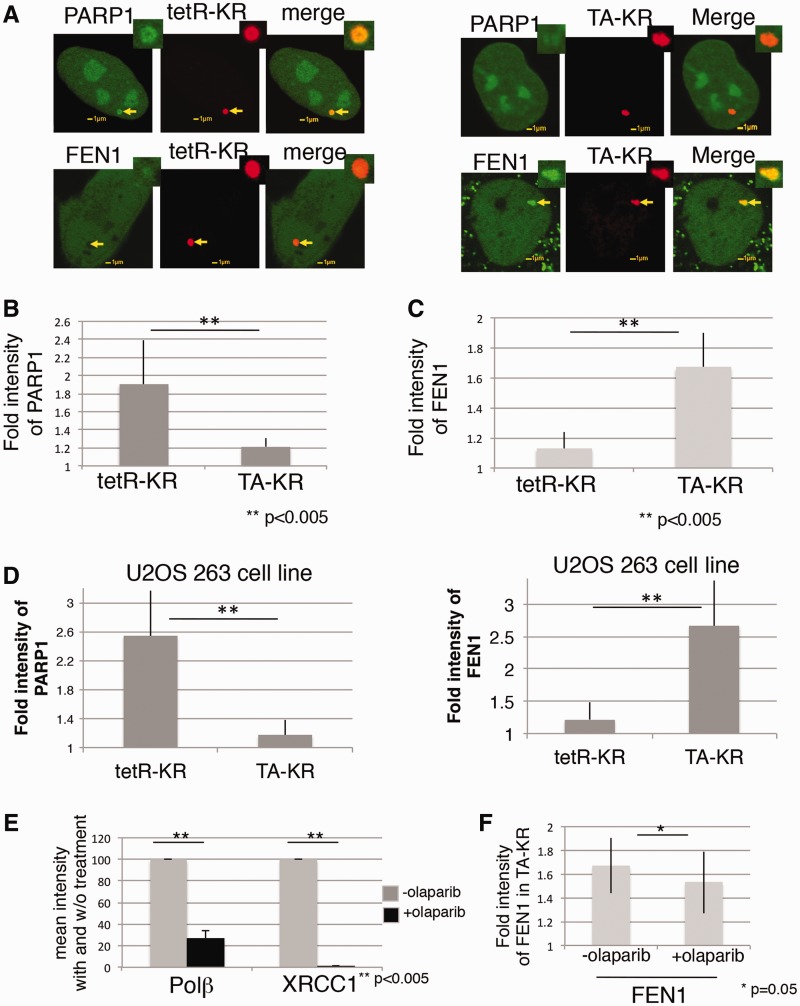
PARP1 is efficiently recruited to the site of tetR-KR but not TA-KR, while FEN1 is recruited to the site of TA-KR but not tetR-KR. (**A**) The damage response of PARP1 (upper panel) or FEN1 (lower panel) in U2OS TRE cells at the site of tetR-KR (left) or TA-KR (right)-induced damage is shown. (**B** and **C**) Quantification of the damage response of GFP-PARP1 (B) and FEN1 (C) at the site of tetR-KR or TA-KR induced damage in U2OS TRE cell line. The fold increase was obtained from foci intensity/background intensity. Mean values with a SD of 10 cells are given in (B), (C) and (D). (**D**) Quantification of the damage response of GFP-PARP1 at the site of tetR-KR or TA-KR induced damage in the U2OS 263 cell line. (**E**) The GFP-XRCC1 or Polß and tetR-KR expressing-U2OS TRE cell line was treated with 10 µM olaparib in medium for 30 min. The damage response of GFP-XRCC1 or Polß was measured 3 min after 559-nm laser bleaching at the site of tetR-KR. The suppression effects of olaparib on the damage response of GFP-XRCC1 and Polß are shown by comparison to the mean intensity of full-length protein w/o olaparib treatment. (**F**) The damage response of FEN1 to TA-KR-induced damage 3 min after 559-nm laser bleaching with treatment of 10 µM olaparib in medium for 30 min. Mean values with a SD in 10 cells are given.

**Figure 7.**
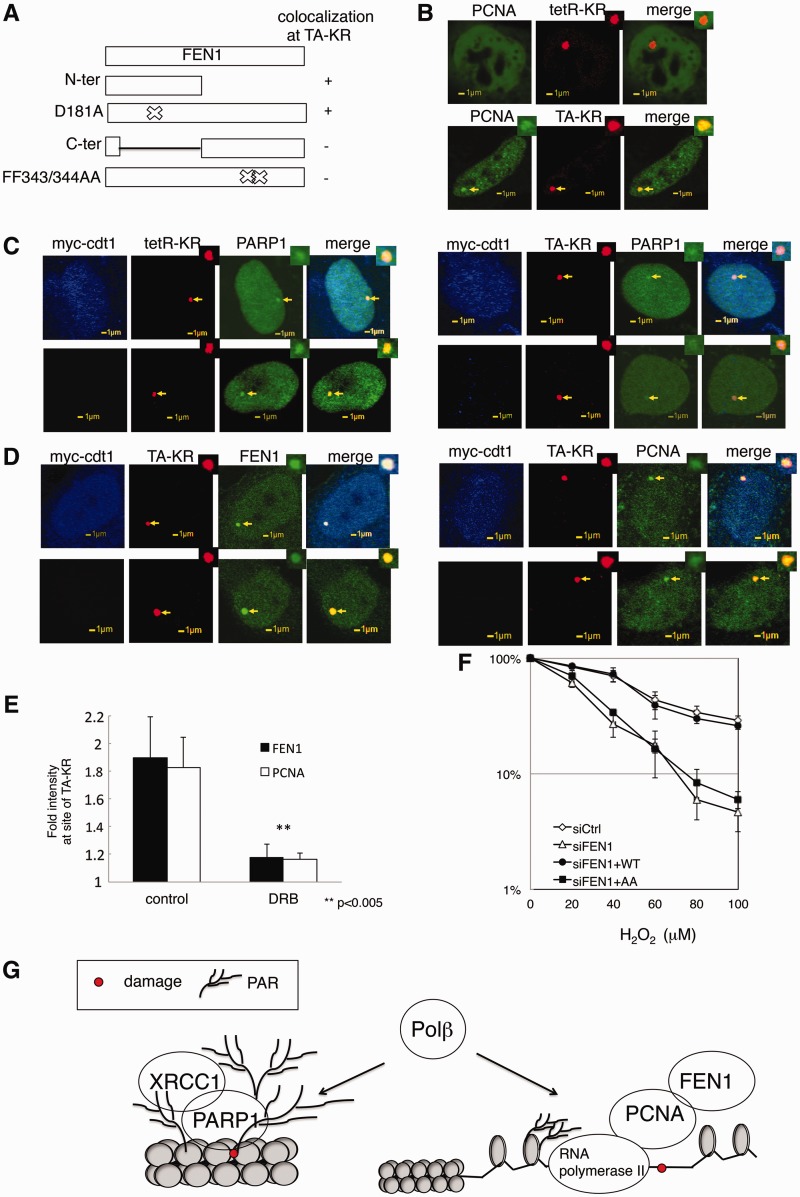
The recruitment of FEN1 to TA-KR-induced damage is dependent on PCNA. (**A**) The damage response of domains and mutations of FEN1 to TA-KR-induced damage 3 min after 559 nm laser bleaching. The N-terminus of FEN1 (N-ter), D184A (flap endonuclease mutant), C-terminus of FEN1 (C-ter) and FF343/344AA (PCNA-binding motif mutation) were used. (**B**) The damage response of GFP-PCNA to tetR- or TA-KR-induced damage 3 min after 559-nm laser bleaching. (**C**) U2OS TRE cells expressing myc-cdt1, GFP-PARP1 and tetR-KR (left) or TA-KR (right). Damage response of PARP1 to the site of tetR (left) or TA (right)—KR-induced DNA damage with exposure to a 15 W SYLVANIA cool white fluorescent bulb for 10 min in cdt1 expressing cells (upper panel) or not expressing cells (lower panel) is shown. (**D**) U2OS TRE cells expressing myc-cdt1, GFP-FEN1 or PCNA and TA-KR. Damage response of FEN1 and PCNA at the site of TA-KR-induced DNA damage with exposure to a 15 -W SYLVANIA cool white fluorescent bulb for 10 min in cdt1-expressing cells (upper panel) or not expressing cells (lower panel) is shown. (**E**) U2OS TRE cells expressing GFP-FEN1, GFP-PCNA and TA-KR are pretreated with or without the RNA polymerase II inhibitor 20 µM DRB in medium for 24 h. The damage response of PCNA and FEN1 at the site of TA-KR-induced DNA damage with exposure to a 15-W SYLVANIA cool white fluorescent bulb for 10 min is shown. (**F**) U2OS TRE cells transfected with sicontrol, siFEN1, siFEN1+ WT-FEN1 or siFEN1+FF343/344AA. A total of 5000 cells were seeded into 96-well plates; 48 h after preparation, cells were treated with H_2_O_2_ in PBC for 1 h at the indicated concentration for the MTT assay. Mean values with a SD for three experiments are given. (**G**) A model of response of repair factors to oxidative DNA damage at condensed chromatin or open chromatin.

The Olympus FV1000 confocal microscopy system was employed (Cat. F10PRDMYR-1, Olympus) and FV1000 software was used for acquisition of images. For bleaching KR, a 559-nm laser was used (FV1000 SIM Scanner set with 405-nm laser diode, Cat. F10OSIM405, Olympus). For inducing DNA damage, a 405-nm laser was used with indicated power; the output power of the 405 laser passed through the lens is 5 mW/scan. Laser light was passed through a PLAPON 60× oil lens (super chromatic abe. corr. obj W/1.4NA FV, Cat. FM1-U2B990). Cells were incubated at 37°C on a thermo-plate (MATS-U52RA26 for IX81/71/51/70/50; metal insert, HQ control, Cat. OTH-I0126) in Opti-MEM during observation to avoid pH changes. For calculation of the percentage of colocalization at the site of KR, 50 cells were counted in every experiment, and representative data are shown (Figures 1D, 2C, D, 3E and 5B). In this case of an identified green spot that colocalizes with the site for KR, the intensity of the green spot at the site of KR compared with the background at other areas is >1.5-fold. Fluoview Soft (Olympus) was used for analysis. In the case of quantification of the DNA damage, the mean intensity of 8-oxoG was measured using Fluoview Soft (Olympus) in cells treated with H_2_O_2_ or light. Mean value of the intensity of 10 cells and ±SD is shown (Figure 2E, Supplementary Figure S2C and D). In most cases of quantification of the damage response of proteins, a ratio of enrichment was used (Figures 2G, 3C, F, 4B, D, 6B–F and 7E). Here, mean intensity of the spot (accumulation of GFP tagged proteins) at the site of tetR or TA-KR/mean intensity of a spot distant from tetR or TA/KR (same size in the same cell) was calculated, and a 1.5-fold increase of intensity was the definition of colocalization. In some cases, the background intensity of GFP expressing protein was subtracted in the same irradiated cell (Figure 3B). The ±SD calculated in each case is shown in the Figure legend. The *P*-value is calculated by Student’s *t*-test using Stat Plus software; *P *< 0.005 is shown as double asterisks.

### KR activation

KR activation was conducted in two ways. Local activation of one KR spot was performed with a 559-nm laser used at the indicated power (1 mW/scan) in a selected area. One scan takes <1 s. Activation of KR in bulk cells was done by exposing cells to a 15-W SYLVANIA cool white fluorescent bulb for 10 min in a stage UVP (Uvland, CA, USA). For calculation of the dose that was delivered to the KillerRed spot, in the case of the 559-nm laser, the laser light was delivered to the selected area (∼25 µm^2^) with 20 mJ/s for 8 s. Therefore, the final power (160 mJ) delivered to the KR (∼1 µm^2^) spot is ∼6 mJ/µm^2^. For calculation of the dose that was delivered to the KillerRed spot based on the pixel size, the pixel size for irradiation is 0.138 µm/pixel and the dwell time per pixel is 8 µs/pixel. The irradiation is at 1.0 mW (1.0 mJ/s). With a dwell time of 8 µs/pixel, this irradiates each pixel with 8.0 nJ/pixel/scan. Multiplying by the number of scans gives the total energy per pixel. In the case of fluorescent light activation, the rate of light is 15 J/m^2^/s. With a 10-min light exposure, 9000 J were delivered to the whole dish; the final power delivered to the KR (∼1 µm^2^) spot is ∼9 mJ/µm^2^ upon light exposure. Cells were placed under a water bottle (height to light is 15 cm) to prevent an increase of temperature.

### Immunoassays and antibodies

Cells in a medium for immunostaining were fixed with methanol-acetone (1:1) for 10 min at −20°C. The fixed cells were dried, then rinsed once with PBS and incubated in blocking buffer (PBS containing the blocking reagent NEN) at 30°C for 30 min. For staining of CPD, 6-4PP and oxo-G, 2.5 N HCL treatment was done to denature DNA before blocking. Cells were then incubated with the first antibody overnight. Cells were washed three times with PBST (PBS with Tween 20) buffer and incubated with Alexa Fluor 405 goat anti-mouse immunoglobulin G, Alexa Fluor 488 donkey anti-goat immunoglobulin G conjugate or Alexa Fluor 488 donkey anti-rabbit immunoglobulin G conjugate (Invitrogen). Cell samples were then mounted in drops of PermaFluor (Immunon). Antibodies used in this research were anti-KR (Ab961, Evrogen), anti-β-actin (sc-1616, Santa Cruz), anti-8-oxo G (dilution 1:50, Millipore MAB3569), anti-γH2AX (1:400, Millipore 05636), anti-poly ADP-ribose (1:100, Millipore MAB3192), anti- H3AcK9 (1:200, Abcam Ab4441), anti-CPD (1:100) and anti-6-4PP (1:100) (provided by Dr. Mori), anti-4H8 (Abcam, 1:400), anti-FEN1 (Santa Cruz, 1:500) and anti-PARP1 (Santa Cruz, 1:500) at the dilution condition recommended by the manufacturer in blocking buffer overnight at 4°C.

## RESULTS

### Chromatin status at site-specific genomic DNA: targeting tetR-KR or TA-KR to an integrated TRE site

To visualize DNA repair transactions with high spatial resolution, damage must be initiated at discrete genetic loci. KR facilitates the formation of oxygen radicals and superoxide through the excited chromophore (15,16) to induce DNA damage. To induce ROS-mediated damage at a specific locus in the genome, we fused KR to the tetracycline repressor to induce ROS damage in a 90-kb tetracycline response element (TRE) array (total of 96 repeats) in U2OS cells (20). Figure 1A shows the scheme for inducing damage by tetR-KR and TA-KR followed by visible light irradiation in U2OS/TRE cell. In the designed plasmid, the I-SceI enzyme recognition site is adjacent to TRE as a random repeat, with a total of 96 repeats. To monitor the status of transcription, the plasmid we utilized contains 24 tandem MS2 bacteriophage viral replicase translational operators (a 19-nt RNA stem loop; MS2 repeats). The transcribed RNA encodes cyan fluorescent protein (CFP) with a peroxisomal targeting signal-1 [Ser-Lys-Leu (SKL)] fused to its carboxyl terminus (Figure 1A). The design of the plasmid was based on a previous study of the visualization of active transcription (21). This plasmid was integrated into a genome site at ∼200 copies (20) and the chromatin status at the site of the integrated plasmid was clarified using a FISH assay to define the precise genomic site of tetR-KR or TA-KR binding in the U2OS/TRE cell line. The integration site of the TRE array was defined as adjacent to the centromere of the X-chromosome (Figure 1B). Staining with DAPI showed a region of relatively condensed DNA at this repeated array (Figure 1B, lower panel) as well as a highly condensed centromere, indicating that the TRE array exists in a heterochromatinized region. With a protein–DNA interaction through TRE and tetR (24), mcherry or KR is bound at the TRE-integrated site of the genome.

To examine the recruitment of DNA repair proteins to sites of DNA damage in regions of active and inactive chromatin, which heretofore was experimentally intractable, we also fused KR to the transcription activator tetR+VP16 (TA) to yield the fusion protein TA-KR (Figure 1A, right). VP16 activates transcription, resulting in the opening of condensed chromatin (25). Since the GFP-MS2 can bind to the stem loop of the translational operator, therefore the synthesized RNA will be visualized (21). Expression of the MS2 coat-YFP fusion protein (MS2-YFP) allows the transcribed RNA to be visualized. When the mRNA is exported from the nucleus and translated in the cytoplasm, the CFP-SKL protein product is targeted to peroxisomes and serves as a reporter, confirming that all processes required for gene expression have been successfully completed. After the CMV promoter, there are the SKL tagged CFP and MS2 repeats which can be used for monitoring the transcription activation (Figure 1A and C). We have monitored the effects of tetR and TA using 5-ethynyluridine (EU) incoporation (to monitor the RNA synthesis), MS2 detection, and expression of CFP at SKL (Figure 1C). As shown in Figure 1C, when TA is expressed at the TRE site, we can monitor the incorporation of RNA and detect MS2 colocalized at the mcherry marked site and CFP expression at SKL. However, no signals could be detected at the site of tetR-mcherry, indicating that transcription is specifically on at the site of TA-mcherry.

The expression of tetR-KR was confirmed by western blot (Supplementary Figure S1A). Three-dimensional reconstruction confocal images show that both tetR-KR and TA-KR form a round sphere of ∼1 µm in diameter together with GFP-HP1α (Supplementary Figure S1B) merged with DAPI, showing that expression of KR is highly localized within one genomic region. Three-dimensional reconstruction confocal images and the actual sizes of XY diameters for tetR-KR and TA-KR regions were measured at the maximum size (Supplementary Figure S1C and D). It appears that the diameter of TA-KR spots in the widest dimension are ∼20% larger than tetR-KR (Supplementary Figure S1D), however this did not reach statistical significance. This apparent increase in size is reasonable since transcription activation will induce chromatin relaxation; therefore the TRE array might diffuse to a larger area compared with that of tetR (21). However, the size of TA-KR does not change significantly, indicating that the transcription units are assembled into a repetitive substructure within the integrated TRE array [(21) and see below].

To further confirm the status of active transcription upon binding of TA-KR, we have stained the site of KR by RNA polymerase II antibody 4H8, which specifically recognizes phosphoserine-5 of the CTD heptapeptide of polymerase II. Phosphorylated RNA Pol II is specifically stained at the site of TA-KR but not tetR-KR before light activation (Figure 1E), confirming that transcription is efficiently and specifically activated at the site of TA-mcherry and KR. Recruitment of YFP-tagged RNA polymerase II (YFP Pol II) was also measured. We also fused monomeric cherry fluorescent protein (mcherry) to tetR and TA as controls. YFP Pol II is specifically recruited to the site of TA-KR as well as TA-mcherry, but not to tetR-mcherry or tetR-KR (Supplementary Figure S1E). The quantification graph with positive foci of 4H8, YFP Pol II that co-localizes with either tetR or TA-KR is shown in Figure 1F. Almost 100% of 4H8 were colocalized with KR at the site of TA, suggesting that the transcription units are active at the site of TA spots. This result indicates that transcription is efficiently and specifically activated with the expression of TA.

To further evaluate the changes in chromatin which is transcriptionally activated via TA-KR expression, we expressed GFP-HP1α in the two systems. GFP-HP1α is highly recruited at the site of tetR, but only shows very slight recruitment at the site of TA (Figure 1E), indicating tetR-KR binds to the TRE in a region of heterochromatin. Above 3D staining of HP1α at the site of KR confirmed this result (Supplementary Figure S1B). Immunostaining of histone H3 acetyl-K9 (AcK9H3) in tetR-KR or TA-KR transfected cells was performed to confirm the chromatin status. In contrast to HP1α, H3AcK9 is enriched at the site of TA-KR but not tetR-KR (Figure 1E), indicating that the chromatin is open at TA-KR (Figure 1E). Magnification of the merged images of HP1α and AcK9H3 with the KR red spot and the quantification of intensity of HP1α and AcK9H3 at the site of tetR and TA (Figure 1F) indicates that the chromatin status at tetR is relatively condensed versus open at TA-KR, corresponding to the previous observations of chromatin states at the site of TA (21).

### Site-specific genomic DNA damage by targeting tetR-KR or TA-KR to an integrated TRE site

KR induces ROS damage by generating superoxide, upon light irradiation (26,27). To understand the nature of tetR-KR induced damage at one genome locus, we illuminated cells with a 15-W Sylvania cool white fluorescent bulb for 10 min and stained cells with antibodies against various DNA damage markers to confirm that only oxidative DNA damage has been induced at the site of tetR-KR binding and localization. The markers of DNA SSBs, PAR and oxidative DNA damage, 8-oxoG, were detected at the sites of both tetR and TA-KR localization after light exposure (Figure 2A and B), showing the production of oxidative DNA damage. We have counted 50 cells to define the colocalization of PAR or 8-oxoG with tetR or TA-KR. After light activation, the frequency of cells showing this colocalization significantly increased by ∼10-fold (Figure 2C and D), showing that induction of oxidative DNA damage is at almost equal frequency at the sites of tetR and TA-KR after light activation.

The UVC photoproducts CPD (cyclobutane pyrimidine dimers) and 6–4PP (6–4 photoproducts) are not detected (Supplementary Figure S2A). GFP-DDB2, a protein that specifically binds to UV dimers even in a heterochromatin structure both *in vitro* and *in vivo* (28,29), is not recruited to the sites of tetR and TA-KR, supporting the conclusion that UVC induced photoproducts are not induced by KR plus light activation. DDB2 is functional since we observed its efficient recruitment to local UVC irradiation damage colocalized at the site of CPD staining (Supplementary Figure S2B). However, XPC, which plays an important role in nucleotide excision repair and binds distorted DNA structures, is recruited at sites of tetR and TA-KR-induced damage (Supplementary Figure S2B). This result is consistent with a previous observation that the binding of XPC is not specific to UVC-induced DNA damage but to a broader spectrum of damage including DNA structure distortions (30,31). More importantly, a recent study shows that XPC can also respond to oxidative DNA damage (32), suggesting a basis for recruitment of XPC at the sites of KR-induced damage.

After showing the production of DNA damage, we tried to quantify the KR levels of 8-oxoG following exposure of the same cell line to various doses of hydrogen peroxide (H_2_O_2_). We have stained 8-oxoG after 1 h of H_2_O_2_ treatment at indicated doses ranging from 0 to 400 mM in PBS for 1 h; the intensity of 8-oxoG compared with the control level (without treatment) was quantified. Cells undergo apoptosis when treated with concentrations of H_2_O_2_ > 400 mM. The intensity of 8-oxoG staining at the spot of KR equals that after 110 mM H_2_O_2_ treatment (equals around 1 µM hydrogen peroxide at the site of tetR-KR) (Supplementary Figure S2C).

As the generation of ROS by KR after light irradiation is a dynamic reaction in cells, in the process superoxide dismutates to H_2_O_2_ and oxygen. Peroxynitrite is also a major product of the diffusion-controlled reaction of superoxide radicals. To test if the damage production is indeed induced by ROS at the site of KR activation, we used ROS scavengers NAC (*N*-acetylcysteine) and/or MnTBAP. NAC is a scavenger for H_2_O_2_ and MnTBAP is a scavenger for peroxynitrite, and both H_2_O_2_ and peroxynitrite are the products of superoxide. NAC (*N*-acetylcysteine) and/or MnTBAP treatment prevents the production of 8-oxoG after H_2_O_2_ treatment (Supplementary Data S2D), indicating that the ROS reaction was inhibited. As after H_2_O_2_ treatment, the production of 8-oxoG at the site of KR was reduced to half level (Figure 2E), supporting that the damage induced by KR after light results from ROS attack.

In cells without exposure to high dose X-ray irradiation, accumulation of oxidative DNA damage is the major reason for the induction of DNA double-strand breaks (DSBs). To understand if DNA DSBs have been induced by KR activation, we next measured the accumulation of γH2AX in the two systems. Both the frequency and the intensity of γH2AX foci at the sites of tetR or TA-KR are similar 30 min after light activation, indicating that the production of damage is similar (Figure 2F) and that DSBs are induced directly or indirectly by KR activation. NAC and MnTBAP treatment diminished the intensity of γH2AX, indicating that production of γH2AX is an outcome of the ROS reaction (Figure 2G). The induction of γH2AX was further confirmed with a ChIP assay at the site of tetR-KR (Supplementary Figure S2E). We detected the fragment around the TRE region in the tetR-KR expressing cells with exposure to light for 10 min, pulled down by γH2AX Ab. Since the γH2AX signals spread to a long distance from the site of a DNA DSB [at least >3 kb (33,34)], detection of γH2AX should be efficient after damage.

### Recruitment of DNA glycosylases to the tetR-KR in heterochromatin and TA-KR in EU-chromatin at one genome locus

Having shown production of base damage and SSBs by KR after light activation (Figure 2A–D), we first assessed if DNA glycosylases are recruited to oxidative damage induced by tetR-KR or TA-KR and light. Since the KR-TRE array can produce highly localized damage, we wanted to increase the temporal resolution of damage by exposure to pulses of laser light. To activate KR, we exposed both tetR-KR expressing (the center spot) and nonexpressing areas (the extended area of the red spot) (rectangle shown in Figure 3A) to 559 nm laser light for 50 scans (over a total of 10 s) at a power rate of 1 mW/scan (equal to 50 mW). After laser light exposure, GFP-NTH1, which recognizes and binds to ROS-damaged DNA bases such as thymine glycol (35), was colocalized only at the site of tetR-KR, but not in an equivalently laser exposed area that does not express tetR-KR (Figure 3A, left). Therefore, the recruitment of GFP-NTH1 did not result from DNA damage induced by the 559-nm laser, but required KR activation induced DNA damage. The same recruitment was also observed in cells expressing TA-KR under the same conditions (Figure 3A, right). This result further suggests that both tetR and TA-KR induced oxygen radicals do not diffuse and are limited to the TRE array. A real-time quantification of the mean intensity of the signals from GFP-NTH1 and tetR-KR is shown in Figure 3B. After photobleaching, the tetR-KR signal recovers with time, indicating that the amount of DNA damage that is produced does not prevent efficient binding of the tetR-KR protein. While the recruitment of GFP-NTH1 reaches its maximum at 3 min and decreases thereafter, almost all of the GFP-NTH1 dissociates from the KR-induced damage site 15 min after irradiation. Increasing exposure to laser light with 70 mW does not induce more recruitment of NTH1 since tetR-KR has been completely bleached (Figure 3C); therefore, the expressed KR could reach its maximum for inducing DNA damage following complete bleaching at certain doses of 559 nm laser light (70 mW). DNA glycosylases NEIL1, NEIL2 were also recruited to both the sites of tetR-KR and TA-KR after laser light activation (Figure 3D). The recruitment of NEIL1 and NEIL2 at the site of tetR and TA-KR after white light activation was quantified (Figure 3E). As after H_2_O_2_ treatment, the recruitment of NTH1 at the site of tetR-KR was reduced to less than half level after NAC and MnTBAP treatment (Figure 3F), supporting that the damage induced by KR after light results from ROS attack.

We next tested the uracil DNA glycosylase MED1 (also known as methyl-binding protein 4, MBD4) and SMUG1. Interestingly, we found that MED1, but not SMUG1 is recruited to both tetR and TA-KR induced damage (Supplementary Figure S3). It is known that MED1 can remove thymine glycol (Tg) mismatched to G resulting from oxidative deamination of 5-methylcytosine (36,37), supporting the hypothesis that a spectrum of oxidative base lesions including Tg:G mispairs, but not uracil, are induced after tetR-KR plus light.

### The 559-nm laser activated tetR-KR initiates BER protein recruitment more efficiently than 405-nm laser microirradiation

Many studies in which the damage response has been analyzed, especially the response to DNA DSBs, have used laser light to induce local DNA damage (38). Attempts to use laser light to induce base damage have been limiting since the physical power of the laser yields only a very small amount of base damage with high-dose irradiation in a background of a large amount of DNA strand breaks (4,39). Here we have compared the recruitment of the DNA glycosylase NTH1 and the BER/SSBR scaffold protein XRCC1 to tetR-KR or 405 laser-induced DNA damage. TetR-KR was activated by the 559-nm laser, while another region within the same cell was irradiated with a 405-nm laser, both with a final power of 50 mW. NTH1 responded to tetR-KR-induced damage, but not to the 405 laser-induced damage (Figure 4A and B), showing preferential BER protein recruitment to the tetR-KR-mediated DNA damage as compared with the damage induced by the 405-nm laser light. By increasing the power of the 405-nm laser 10-fold, NTH1 showed a similar intensity at the site exposed to the 405-nm laser when compared with the tetR-KR-mediated DNA damage (Figure 4A and B).

XRCC1 is efficiently recruited to the DNA damage induced by the 405-nm laser, yet it is less efficiently recruited to the site of tetR-KR-induced damage (Figure 4C and D). The recruitment of XRCC1 following DNA damage induced by the 405-nm laser is >5-fold than the level of XRCC1 recruitment to the tetR-KR-induced damage (Figure 4B, right). With the same 405-nm laser dose, the DNA glycosylases NTH1 (Figure 3A) and NEIL1 or NEIL2 (not shown) are not recruited. Although a variety of glycosylases have been recruited (Figure 3) to the site of tetR-KR induced damage to initiate BER, this did not cause an additive response of XRCC1, suggesting that XRCC1 plays a limited role in BER for the repair of tetR-KR-induced DNA damage compared with its role in the repair of SSBs. This is not unexpected since BER can be completed by long-patch repair synthesis, not completely dependent on XRCC1 (40).

Next we sought to define the domains of XRCC1 that are required for recruitment to DNA damage induced by activated tetR-KR. To this end, we measured the recruitment of XRCC1 truncated proteins in response to DNA damage from 405-nm laser induction or tetR-KR activation. The BRCT I domain of XRCC1 was required for recruitment, whereas neither the NTD (1–300 aa) nor the BRCT II domain alone was sufficient to promote recruitment after either 405 nm laser-induced or KR-induced damage (Figure 4E and Supplementary Figure S4A). The BRCT I domain is recruited to tetR-KR induced DNA damage and since the BRCT I domain alone is sufficient for the recruitment of XRCC1, we used the XRCC1 BRCT I domain mutant (LI360/361DD), which abolishes its binding to PAR (5), and found that the LI360/361DD mutant did not respond to KR-induced damage (Figure 4E, LI360/361DD). These results suggest that the recruitment of XRCC1 at the site of tetR-KR damage occurs through PARP activation.

### Recruitment of DNA Polß to the site of tetR and KR is damage dependent and genome site independent

Having shown that BER is more efficiently initiated in both the tetR-KR and TA-KR systems, with the recruitment of glycosylases (Figure 3), than 405 laser microirradiation (Figure 4), we next measured the recruitment of GFP-Polß and found that it also localized at the sites of both tetR and TA-KR-induced damage (Figure 5A), suggesting an active site undergoing DNA repair. This recruitment is the result of KR-induced ROS and not by a nonspecific interaction or recruitment of Polß to the TRE since Polß is not recruited to tetR and TA-mcherry, but specifically localizes with tetR-KR and TA-KR after light exposure (Figure 5A and B). This result demonstrates that the interaction between TRE and tetR does not cause nonspecific binding of repair factors and that Polß recruitment is specific for the ROS damage induced by activated KR.

To exclude the possibility that this recruitment happens only at a specific region (centromere) of the X-chromosome in the U2OS TRE cell line, we used another U2OS cell line (U2OS/263) with similar copy numbers of integrated TREs at chromosome 1p36 near the telomere (21). This cell line also forms a heterochromatin structure. GFP-Polß was recruited to the site of tetR-KR in the U2OS/263 cell line as well as in the U2OS/TRE cell line, but at a different location in the genome (Figure 5C), indicating that this is not a response specific to the U2OS/TRE cell line at the X-chromosome.

### PARP1 is efficiently recruited to the site of tetR-KR while FEN1 is efficiently recruited to the site of TA-KR

Having validated the use of the KR system to efficiently induce BER protein recruitment, we next wanted to examine the recruitment of downstream DNA repair proteins to sites of DNA damage in regions of active and inactive chromatin. We wished to understand the role of PARP1 activation in the repair of oxidative DNA damage within different chromatin structures, and for this we studied the localization of GFP-PARP1 at the tetR-KR and TA-KR damage sites (Figure 6A, left panel). Although PARP1 is recruited to the site of TA-KR, the recruitment of PARP1 was much stronger at the site of tetR-KR compared with TA-KR (Figure 6A and B), indicating that PARP1 might play a more important role for repair in a condensed chromatin structure compared with an open chromatin structure. Interestingly, recruitment of FEN1, which is involved in the long patch subpathway of BER (41), was much more efficient at damaged sites of TA-KR, as compared with tetR-KR damage sites (Figure 6A, right panel and C).

Next, to further confirm that this is not due to the specific site of integration in our U2OS TRE cell line, we used the U2OS/263 cell line (21) (TREs at chromosome 1p36 near the telomere with a heterochromatin structure) again to test this conclusion. The recruitment of PARP1 and FEN1 in the U2OS/263 cell line showed a similar trend (Figure 6D), indicating that this was not due to the specific integrated site in the U2OS TRE cell in the X-chromosome and that the recruitment of PARP1 and FEN1 at different genome loci might share a common mechanism.

To confirm the role of PARP activation in heterochromatin, the kinetics of Polß and XRCC1 recruitment were quantified in the tetR-KR system. With the addition of olaparib, a PARP1 inhibitor (PARPi), the recruitment of Polß was decreased to 35% (Figure 6E) as compared with vehicle control, and the recruitment of XRCC1 was completely abolished (Figure 6E). Another inhibitor, PARPi PJ34, confirmed the effects (Supplementary Figure S4B). Both inhibitors are functional since 30 min treatments of cells completely suppressed the recruitment of XRCC1 to 405-nm laser induced DNA damage (Supplementary Figure S4B). Furthermore, PARPi treatments did not alter the cell cycle progression compared with untreated cells (Supplementary Figure S4C). We further confirmed the effects of PARPi by using the siPARP1. siPARP1 suppressed the recruitment of Polß at the site of tetR-KR but did not show much effect on the recruitment of FEN1 at the site of TA-KR (Supplementary Figure S5A). Polß is recruited both at the site of tetR and TA-KR efficiently, consistent with previous results showing that 8-kDa N-terminal Polß is recruited to base damage independently of XRCC1 (4). The 8 kDa Polß 5′dRP N-terminal domain lyase might respond to KR-induced damage mediated by a direct interaction with the deoxyribose moiety, which is generated during BER (42). This is in line with the complementation of Polß KO cells with the 8-kDa domain of Polß (43). We next examined the recruitment of FEN1 at sites of TA-KR-induced damage. The recruitment of FEN1 to sites of TA-KR induced DNA damage is not efficiently affected by treatment with the PARPi olaparib (Figure 6F). This is to be expected since PARP1 itself is not enriched at the site of TA-KR (Figure 6F).

### Recruitment of FEN1 to DNA damage in active chromatin is dependent on PCNA

To understand how FEN1 is recruited to the site of TA-KR, we further examined the recruitment of FEN1 deletion or point mutants to sites of TA-KR induced damage. The recruitment of FEN1 is not induced by structural changes at sites of TRE after binding with TA, since FEN1 could not be recruited to sites of TA and tetR when they were fused with mCherry (Supplementary Figure S5B). Both the N-terminal domain of FEN1 (N-ter) and the D181A FEN1 mutant, which abolishes its flap endonuclease activity (44), are recruited to the site of TA-KR induced damage. PARPi treatment did not abolish the recruitment of N-ter, D181A FEN1 or wild type FEN1. However, the C-terminal domain of FEN1 (C-ter) and the double mutant of FEN1 (FF343/344AA), which abolishes its interaction with PCNA (45) were not recruited to the sites of TA-KR induced damage (Figure 7A and Supplementary Figure S5B). In support of this result, we found that PCNA is specifically localized to sites of TA-KR-induced DNA damage, but not to sites of tetR-KR-induced damage (Figure 7B). Therefore, the recruitment of FEN1 to DNA damage at sites of transcriptionally active chromatin is dependent on PCNA.

To understand if the recruitment of FEN1 at TA-KR is a late-S phase specific response, we costained the G0/G1 cell-cycle marker cdt1 (46,47) with FEN1 and TA-KR. As shown in Figure 7C and D, both PARP1 and FEN1 is co-localized at the site of TA-KR after light activation, in either cdt1 stained (G1) or nonstained (S/G2) cells, indicating that recruitment of FEN1 to DNA damage in active chromatin is dependent on PCNA; both FEN1 and PCNA are recruited to KR plus light induced DNA damage not only in late-S phase but also in G0/G1 phases.

To understand if the transcription processes affect the recruitment of FEN1, we checked the effects of the RNA polymerase II inhibitor, 5,6-dichloro-1 -β-d-ribofuranosylbenzimidazole (DRB). Interestingly, DRB treatment diminished the recruitment of FEN1 at the site of TA (Figure 7E), indicating that an active transcription machinery/open chromatin state is necessary for the efficient recruitment of repair factors involved in long-patch repair. Finally, we tested if the double mutant of FEN1 (FF343/344AA), which could not respond to KR+light-induced DNA damage, could rescue the sensitivity of cells to oxidative DNA damage. We used siRNA targeting the UTR region of endogenous FEN1, and expressing either WT or MT (FF343/344AA) FEN1 in U2OS cells. After treating cells with H_2_O_2_ at indicated concentrations, the WT but not MT rescues the cell’s sensitivity to H_2_O_2_ (Figure 7F), indicating that the damage response of FEN1 is necessary for cell survival after oxidative DNA damage. A model of the damage response of repair factors to oxidative DNA damage at condensed chromatin or open chromatin is shown in Figure 7G. Our results indicate that although production of DNA damage and recruitment of DNA glycosylases and Polβ at the sites of tetR-KR (transcription off) and TA-KR do not show a difference, the recruitment of PARP is much more efficient in condensed chromatin. In contrast, the recruitment of FEN1 is dependent on PCNA, while the recruitment of PCNA is further dependent on active transcription (Figure 7G). Taken together, these results suggest that the open chromatin already provides space for the LPR subpathway of BER. Our results presented here indicate the importance of chromatin and transcription status in the selection of subpathways of BER, and the histone and protein modifications at the site of tetR-KR (OFF) and TA-KR (on) that affect the choice of repair pathways will be further identified in future work.

## DISCUSSION

In this study, we established a novel system for the induction of oxidative DNA damage at high spatial and temporal resolution using KR fused to tetR or TA. Using this system, we show that ROS damage is formed within seconds of laser light activation at a specific region on the X-chromosome in regions of condensed or open chromatin. We characterized whether the DNA glycosylases NTH1, NEIL1, NEIL2, MED1 and SMUG-1, as well as the DNA repair factors PARP1, XRCC1, DNA Polß and FEN-1, are recruited to these damaged sites.

The FISH assay indicates that the TRE array exists in a heterochromatinized region (Figure 1B). Here, we found that using a fluence of the 559-nm laser at 50 mW focused on the tetR-KR locus induced 8-oxoG lesions (Figure 2). Furthermore, we found that many DNA glycosylases (NTH1, NEIL1 and NEIL2) were efficiently recruited to the site of tetR-KR-induced damage (Figure 3). The tetR-KR system is a powerful tool to induce base damage and initiate BER at a specific genome site, unlike the 405-nm laser which generates a high background of strand breaks and induction of heat shock proteins (4). KR had been thought to generate singlet oxygen, and more recently was shown to induce superoxide radical anions following light irradiation (26,27). Using our site-specific tetR-KR damage array approach, we were able to observe differential recruitment of DNA repair proteins, which was dependent upon the specific chromatin status. The 405-nm laser system was not capable of showing this differential recruitment. In addition to inducing DNA damage at specific genome sites, the tetR-KR system has several other advantages, e.g. it can be used for measuring the effects of DNA damage on cell function, both with single cell assays and cell population assays when KR is activated by a 559-nm laser or total dish illumination with white light, respectively.

We have assessed the recruitment of proteins in a specifically defined chromatin structure using KR fused to either tetR or TA. We have detected effective transcription at sites of TA-KR in U2OS cells, as H3AcK9, a marker of transcriptionally active chromatin, is specifically stained at the site of TA-KR (Figure 1). Therefore the X-chromosome in the U2OS cell line is not transcriptionally inactive as might happen in a female in one of the allelic X-chromosomes (48). Compared with the KR method, micro-irradiation with the 405-nm laser induces DNA damage throughout the cell nucleus and the chromatin status is ambiguous with respect to the various sites of DNA damage. We compared the response of PARP1 and repair factors involving either short patch or long patch BER within condensed or open chromatin structures. PARP1 is more efficiently recruited at sites of condensed chromatin (Figure 6). Furthermore, we showed that the recruitment of XRCC1 and Polß largely depends on PARP activation at sites of condensed chromatin (Figure 6). Although it is well known that PARP will be activated to synthesize pAR at the sites of DNA SSBs, the function of PARP in forming BER intermediates is unclear because the processes are much more complicated and may include AP endonuclease and other processing enzymes. In the heterochromatin structure, to facilitate the recruitment of repair factors, PARP1 might be enriched and activated well. However, at the active transcription unit and open chromatin state, the activation of PARP1 is not as essential as within the heterochromatin. A recent study indicates that FEN1 is recruited to local laser-induced DNA damage sites and this recruitment is partly dependent on PARP1 activation (49). As mentioned earlier, conceptualized in three dimensions, the 405-nm laser irradiates sites from the bottom to the top of a cell. Therefore, the 405 nm laser induces DNA damage randomly with respect to the chromatin and transcription status in the cell nucleus. Our results show that the recruitment of FEN1 is enriched at sites of active chromatin (Figure 6) together with and dependent on PCNA (Figure 7). High Mobility Group Box 1 (HMGB1) has been shown to be a cofactor in the regulation of BER subpathways, inhibiting single-nucleotide BER and stimulating long-patch BER (50). Since HMGB1 is involved in chromatin remodeling and transcription at sites of DNA damage, it might contribute to opening chromatin at transcriptionally active sites, thereby enhancing the recruitment of FEN1. Taken together, these results suggest that the open chromatin provides space for the LPR subpathway of BER. DRB treatment significantly affected the recruitment of FEN1 and PCNA at the TA-KR site (Figure 7E), indicating the possibility that FEN1 and PCNA are necessary for the transcription coupled repair of ROS damage. Furthermore, FEN1 is acetylated by p300 (51), and acetylation in a transcription-active chromatin environment could increase the rate of damaged nucleotide removal during DNA repair. Overall, our results presented here indicate the importance of chromatin and transcription status in the selection of subpathways of BER. How transcription affects the recruitment of BER factors and repair of oxidative DNA damage will be studied with the KR system in future work.

Taken together, our results show that repair proteins are differentially recruited to open or condensed chromatin at specific genetic loci. Future studies will investigate the role of specific chromatin remodeling factors in the repair of damage in these different chromatin states. The fact that KR can be targeted to unique sites in the genome will be of considerable value. It would be interesting to determine if and how site-specific homologous recombination is induced by ROS damage and regulated in different chromatin structures at the site of tetR and TA. Furthermore, KR-induced ROS production mimics the natural condition, i.e. in cells without exposure to high-dose X-ray irradiation, accumulation of oxidative DNA damage would be a major reason for the induction of DNA DSBs. The damage response of proteins involved in different repair pathways would provide the information for crosstalk of different repair pathways at the site of tetR and TA. It is also possible to tag KR with Tfam (52) to induce DNA oxidative damage restricted at a site in mitochondrial DNA, or with TRF1 (53) at a site in telomeric DNA. BER contributes to trinucleotide repeat expansion (54), but how BER proteins are recruited to repeating sequences *in vivo* is not known. Analyzing the recruitment of DNA damage repair proteins to these diverse DNA sites will serve as a foundation for future work examining the mechanisms of oxidative DNA damage and repair throughout the nuclear and mitochondrial genome. Furthermore, this novel system will allow detailed analysis of the effects of site-specific DNA damage and repair on cellular functions.

## SUPPLEMENTARY DATA

Supplementary Data are available at NAR Online.

## FUNDING

The Competitive Medical Research Fund (CMRF) of the University of Pittsburgh Medical Center (UPMC) (to L.L. and S.N.); China Scholarship Council (to L.S.) and grants from the National Institutes of Health (NIH) [AG045545-01 to L.L.; CA148629; GM087798; ES019498; GM099213 to R.W.S.; ES019566 to B.V.H.; and GM103529 to M.P.B.]; Support for the UPCI Imaging Facility and UPCI Cytometry Facility were provided by the Cancer Center Support Grant from the National Institutes of Health [P30 CA047904]. Funding for open access charge: National Institutes of Health [AG045545-01].

*Conflict of interest statement*. None declared.

## Supplementary Material

Supplementary Data
