# Supplementary material for: Novel method for site-specific induction of oxidative DNA damage reveals differences in recruitment of repair proteins to heterochromatin and euchromatin
Source: Nucleic Acids Res. 2013 Nov 29;42(4):2330–45. doi: 10.1093/nar/gkt1233 (PMC3936713; doi:10.1093/nar/gkt1233)
Supplement: Supplementary Data [file supp_42_4_2330__index.html]

Novel method for site-specific induction of oxidative DNA damage reveals differences in recruitment of repair proteins to heterochromatin and euchromatin — Supplementary Data 

# Novel method for site-specific induction of oxidative DNA damage reveals differences in recruitment of repair proteins to heterochromatin and euchromatin

## Supplementary Data

files

**Files in this Data Supplement:**

- Supplementary Data - pdf file
