# Supplementary material for: Novel method for site-specific induction of oxidative DNA damage reveals differences in recruitment of repair proteins to heterochromatin and euchromatin
Source: Nucleic Acids Res. 2013 Nov 29;42(4):2330–45. doi: 10.1093/nar/gkt1233 (PMC3936713; doi:10.1093/nar/gkt1233)
Supplement: Supplementary Data [file supp_gkt1233_nar-02263-d-2013-File009.pdf]

Supplementary Information

Supplementary Figure 1. A. Western blot of KR expression in U2OS TRE cells. **B.** Three dimensional reconstruction (XY axis, YZ and XZ axis) confocal images of tetR-KR and TA-KR merged with DAPI. **C.** The average sizes of XY diameters for tetR-KR and TA-KR regions were measured in 10 cells at the maximum size; SD is the size in 10 cells. **D.** U2OS/TRE cells were transfected with YFP Pol II and tetR-KR, TA-mcherry or TA-KR. 24 hrs after transfection, the images show the recruitment of YFP Pol II 10 min after exposure to the 15 watt SYLVANIA cool white fluorescent bulb for 10 min. **E.** Three-dimensional view of colocalization of HP1α and the site of tetR-KR is shown.

Supplementary Figure 2. A. U2OS TRE cells with tetR-KR and with exposure to the 15 watt SYLVANIA cool white fluorescent bulb for 10 min were stained after treatment with anti-CPD, -6-4PP and anti-KR. **B.** DDB2 is not recruited to the site of either tetR-KR or TA-KR (upper panel) but to local UVC irradiation. XPC is recruited to the site of tetR-KR and TA-KR after 559 nm laser bleaching in U2OS TRE cells (lower panel). **C.** U2OS cells were treated with indicated H_2O_2 concentrations in PBS for 1 hr. After treatment, cells were fixed and stained with 8-oxoG. The intensity of 8-oxoG was measured to make the graph. Staining of 8-oxoG at the site of tetR-KR was measured at the same time in cells with exposure to the 15 watt SYLVANIA cool white fluorescent bulb for 10 min. The intensity is the average of 10 cells. **D.** tetR-KR expressing U2OS TRE cells were treated with NAC (20 mM) or MnTBAP (100 μM) immediately before exposure to a 15 watt SYLVANIA cool white fluorescent bulb for 10 min. Cells were

fixed and stained with 8-oxoG after light activation. Mean values with a standard deviation (SD) of 10 cells are given. **E.** Amplification of the DNA fragment using the ChIP-IT express enzymatic kit. Templates are from the genomic DNA extracted from tetR-KR expressing U2OS TRE cells 10 min after exposure to a 15 watt SYLVANIA cool white fluorescent bulb for 10 min, with or without IP by α–γH2AX.

Supplementary Figure 3. The recruitment of MED1 and SMUG1 to tetR-KR or TA-KR-induced damage with exposure to the 15 watt SYLVANIA cool white fluorescent bulb for 10 min.

Supplementary Figure 4. A. Damage response of XRCC1 to the 405 nm laser (30 ms irradiation)-induced DNA damage with 4 μM PJ34 or 10 μM oraplib, and without the indicated PARP inhibitors in U2OS TRE cells. **B.** Cell cycle progression by FACS with or without indicated PARP inhibitors for 30 min in U2OS TRE cells. **C.** The damage response of XRCC1 and its domains to tetR-KR-induced damage 3 min after 559 nm laser bleaching in U2OS TRE cells.

Supplementary Figure 5. A. Western blot of PARP1 expression in U2OS TRE cells with or without siPARP1 treatment. The recruitment of Polß to tetR-KR and FEN1 to TA-KR-induced damage 3 min after 559 nm laser bleaching is shown. **B.** FEN1 is not recruited to the site of tetR-mCherry and TA-mCherry 3 min after 559 nm laser bleaching in U2OS TRE cells. **C.** The recruitment of domains and mutations of FEN1 to TA-KR-

induced damage 3 min after 559 nm laser bleaching. **D.** Western blot of FEN1 expression in U2OS TRE cells with or without siFEN1 treatment.

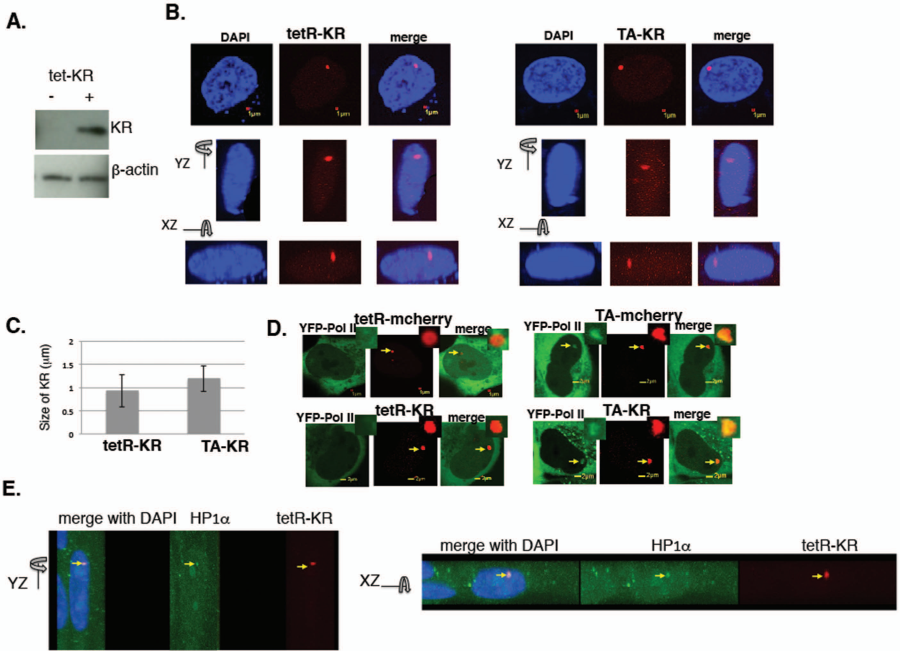
A.
tet-KR
-
+
KR
β-actin
B.
DAPI
tetR-KR
merge
DAPI
TA-KR
merge
1μm
YZ
XZ
C.
Size of KR (μm)
2
1.5
1
0.5
0
tetR-KR
TA-KR
D.
YFP-Pol II
tetR-mcherry
merge
TA-mcherry
tetR-KR
TA-KR
2μm
E.
merge with DAPI
HP1α
tetR-KR

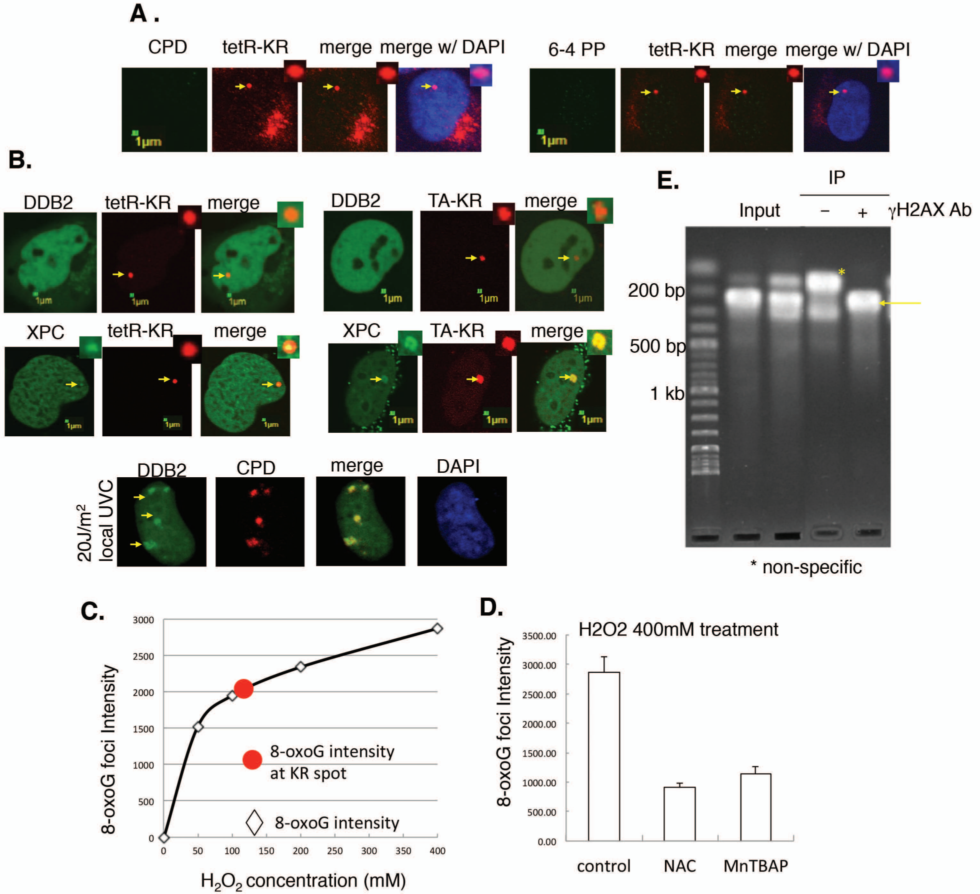
A.
CPD
tetR-KR
merge
merge w/ DAPI
6-4 PP
tetR-KR
merge
merge w/ DAPI
1μm
B.
DDB2
tetR-KR
merge
DDB2
TA-KR
merge
XPC
tetR-KR
merge
XPC
TA-KR
merge
1μm
20J/m2 local UVC
DDB2
CPD
merge
DAPI
E.
IP
Input
−
+
γH2AX Ab
200 bp
500 bp
1 kb
*
* non-specific
C.
8-oxoG foci Intensity
3000
2500
2000
1500
1000
500
0
0
50
100
150
200
250
300
350
400
H2O2 concentration (mM)
8-oxoG intensity at KR spot
8-oxoG intensity
D.
H2O2 400mM treatment
8-oxoG foci Intensity
3500.00
3000.00
2500.00
2000.00
1500.00
1000.00
500.00
0.00
control
NAC
MnTBAP

Supplementary Figure 3

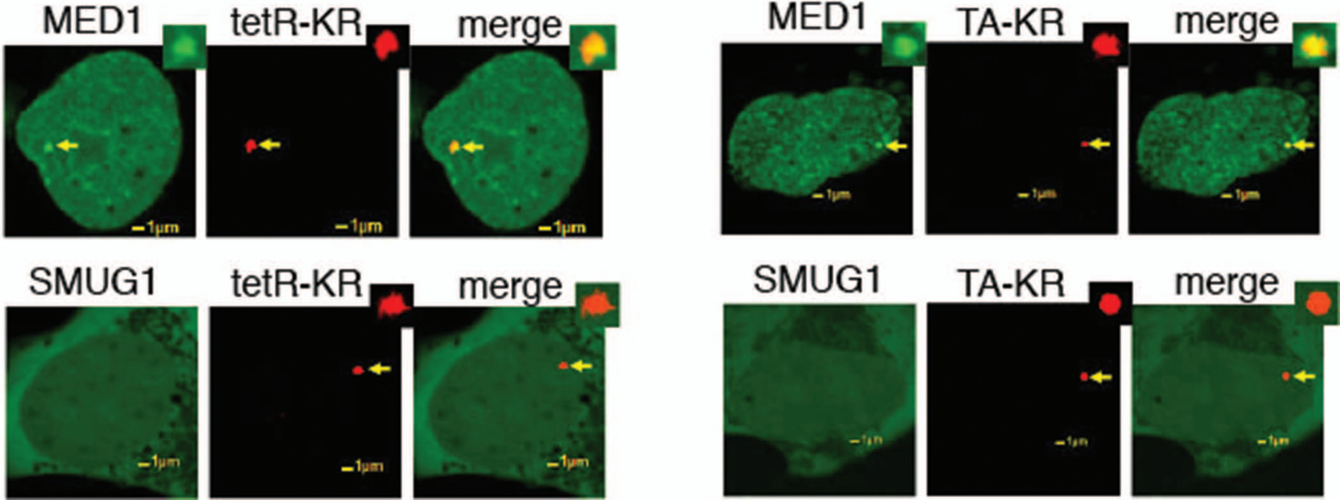

Supplementary Figure 4

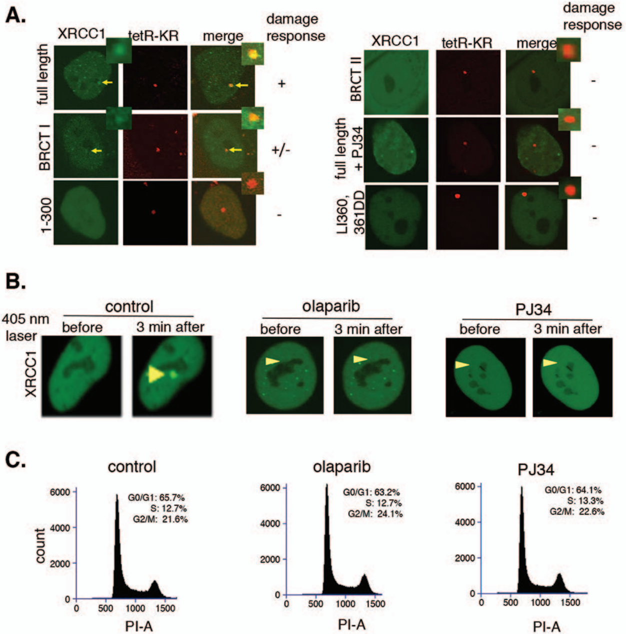

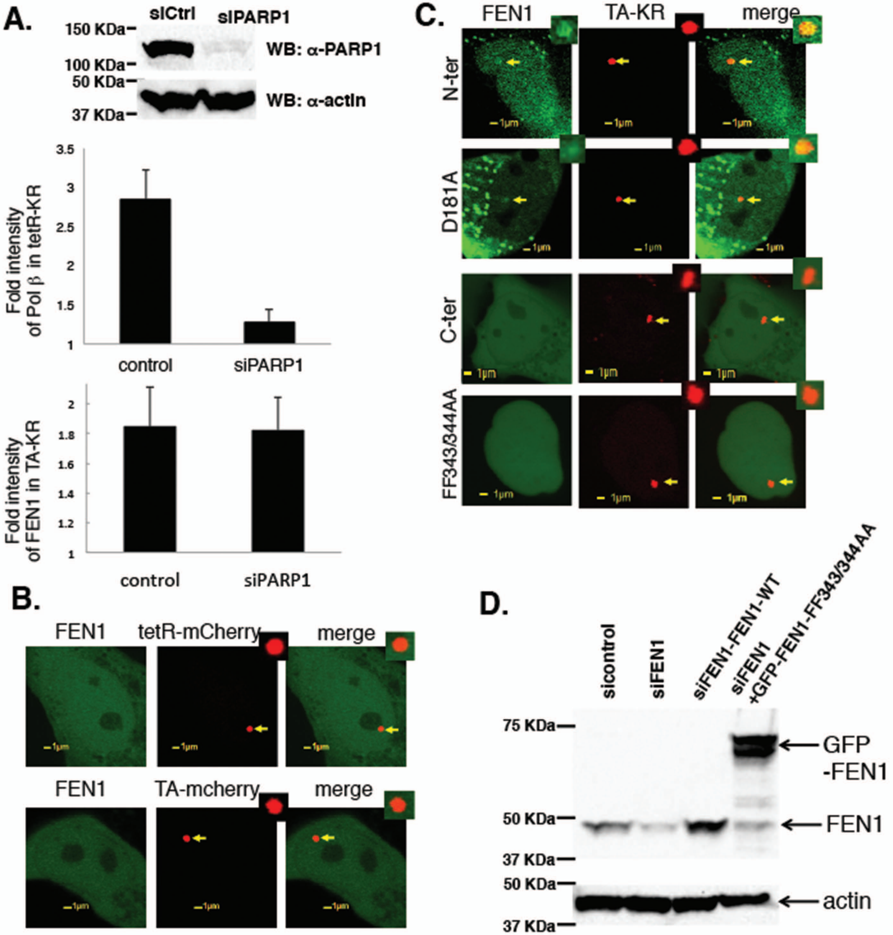

A.
siCtrl
siPARP1
150 KDa
100 KDa
WB: α-PARP1
50 KDa
37 KDa
WB: α-actin
Fold intensity of Pol β in tetR-KR
control
siPARP1
Fold intensity of FEN1 in TA-KR
control
siPARP1
B.
FEN1
tetR-mCherry
merge
FEN1
TA-mcherry
merge
1μm
C.
FEN1
TA-KR
merge
N-ter
D181A
C-ter
FF343/344AA
1μm
D.
sicontrol
siFEN1
siFEN1-FEN1-WT
siFEN1 +GFP-FEN1-FF343/344AA
75 KDa
50 KDa
37 KDa
GFP -FEN1
FEN1
actin
